# Supplementary material for: DMRforPairs: identifying Differentially Methylated Regions between unique samples using array based methylation profiles
Source: BMC Bioinformatics. 2014 May 15;15:141. doi: 10.1186/1471-2105-15-141 (PMC4046028; doi:10.1186/1471-2105-15-141)
Supplement: Additional file 2 — DMRforPairs output for the comparison of A431-MCF7 and NA17018-NA17105. Please start from the HTML files in each folder. Available via the BMC Bioinformatics website. [file 1471-2105-15-141-S2.zip › 1394847754114233_MOESM2_ESM/A431_MCF7/figures/18306.pdf]

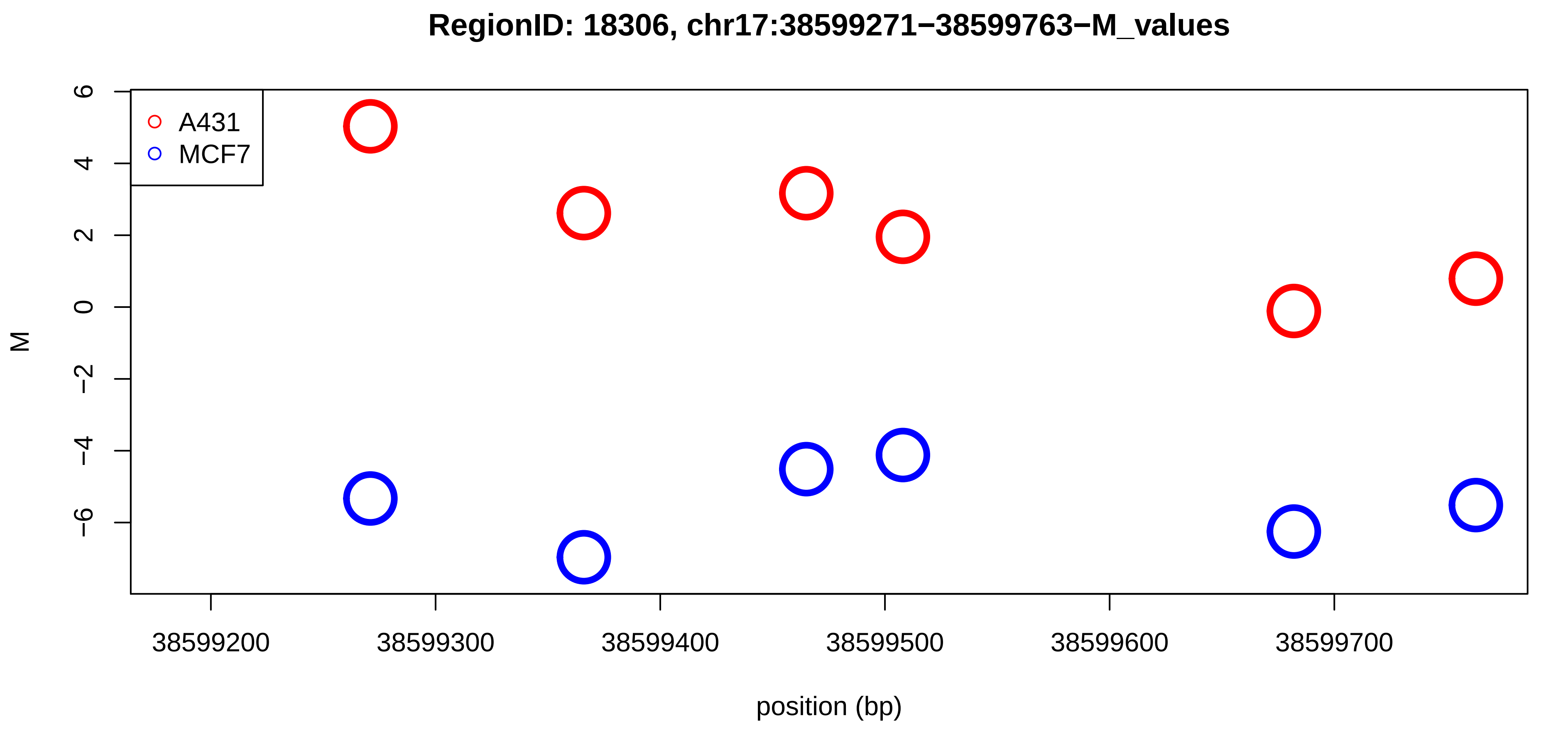
RegionID: 18306, chr17:38599271−38599763−M_values
A431
MCF7
M
position (bp)
38599200
38599300
38599400
38599500
38599600
38599700
6
4
2
0
−2
−4
−6

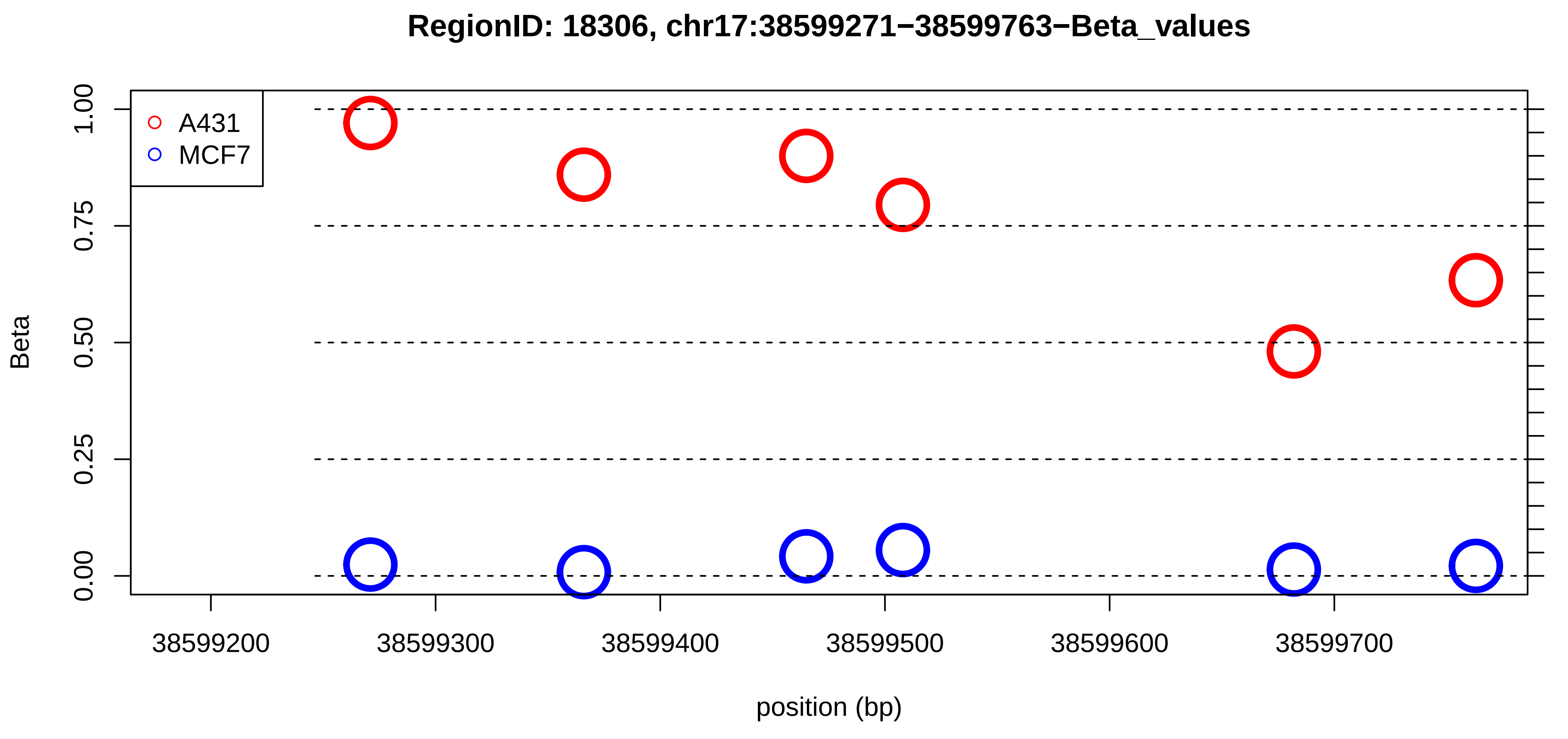
RegionID: 18306, chr17:38599271−38599763−Beta_values
A431
MCF7
Beta
position (bp)
38599200
38599300
38599400
38599500
38599600
38599700
1.00
0.75
0.50
0.25
0.00

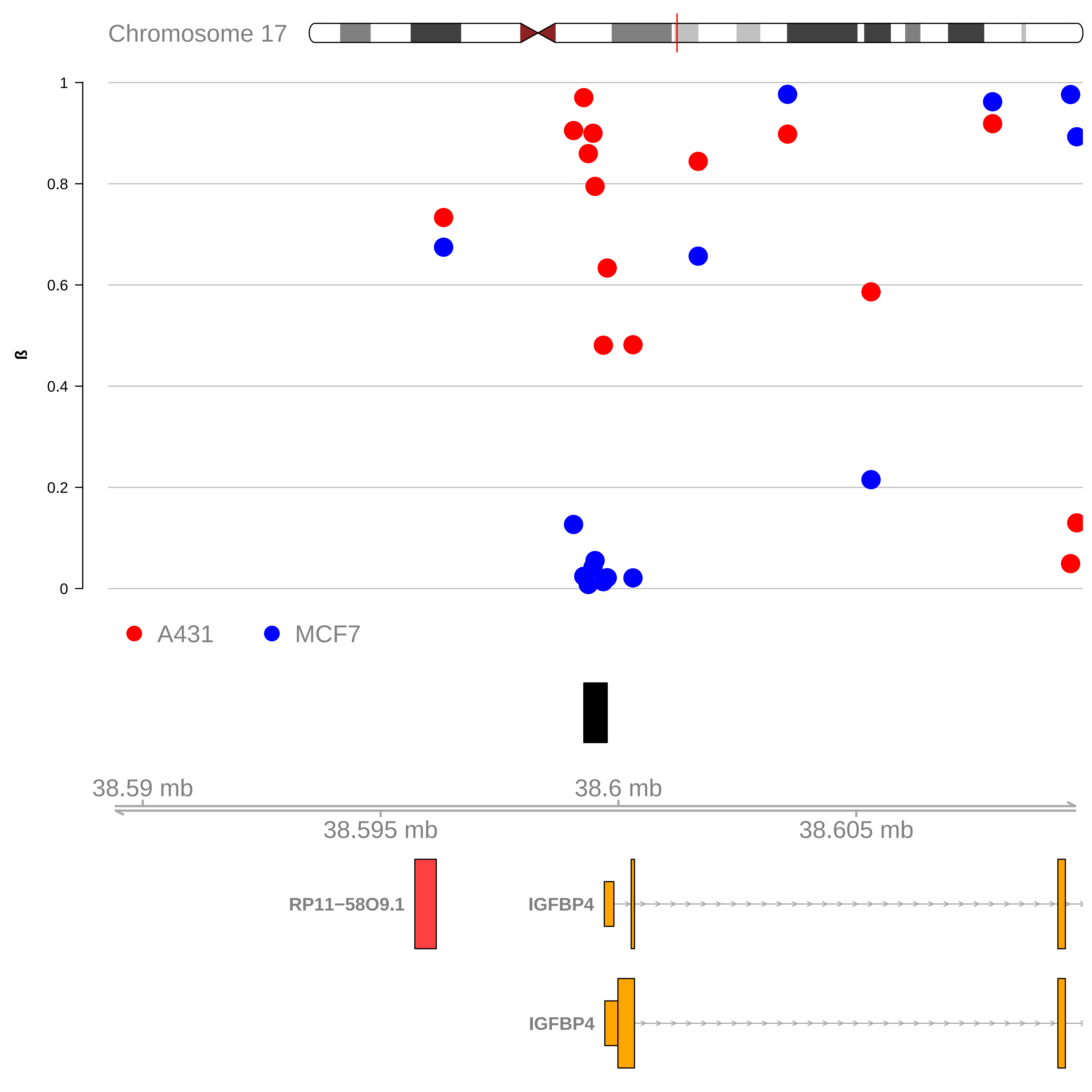

Chromosome 17
1
0.8
0.6
0.4
0.2
0
β
A431
MCF7
38.59 mb
38.6 mb
38.595 mb
38.605 mb
RP11−58O9.1
IGFBP4
IGFBP4
